# Low household income and neurodevelopment from infancy through adolescence

**DOI:** 10.1371/journal.pone.0262607

**Published:** 2022-01-26

**Authors:** Nicole L. Hair, Jamie L. Hanson, Barbara L. Wolfe, Seth D. Pollak

**Affiliations:** 1 Department of Health Services Policy and Management, Arnold School of Public Health, University of South Carolina, Columbia, South Carolina, United States of America; 2 Department of Psychology, University of Pittsburgh, Pittsburgh, Pennsylvania, United States of America; 3 Learning Research & Development Center, University of Pittsburgh, Pittsburgh, Pennsylvania, United States of America; 4 Department of Economics, University of Wisconsin—Madison, Madison, Wisconsin, United States of America; 5 Department of Population Health Sciences, University of Wisconsin—Madison, Madison, Wisconsin, United States of America; 6 La Follette School of Public Affairs, University of Wisconsin—Madison, Madison, Wisconsin, United States of America; 7 Institute for Research on Poverty, University of Wisconsin—Madison, Madison, Wisconsin, United States of America; 8 Department of Psychology, University of Wisconsin—Madison, Madison, Wisconsin, United States of America; 9 Waisman Center, University of Wisconsin—Madison, Madison, Wisconsin, United States of America; Boys Town National Research Hospital, UNITED STATES

## Abstract

Despite advancements in the study of brain maturation at different developmental epochs, no work has linked the significant neural changes occurring just after birth to the subtler refinements in the brain occurring in childhood and adolescence. We aimed to provide a comprehensive picture regarding foundational neurodevelopment and examine systematic differences by family income. Using a nationally representative longitudinal sample of 486 infants, children, and adolescents (age 5 months to 20 years) from the NIH MRI Study of Normal Brain Development and leveraging advances in statistical modeling, we mapped developmental trajectories for the four major cortical lobes and constructed charts that show the statistical distribution of gray matter and reveal the considerable variability in regional volumes and structural change, even among healthy, typically developing children. Further, the data reveal that significant structural differences in gray matter development for children living in or near poverty, first detected during childhood (age 2.5–6.5 years), evolve throughout adolescence.

## Introduction

Early clinical applications of brain development employed basic measures, such as measuring head circumference growth curves, to identify children in need of medical follow-up [1]. Over time, the improved quantification of both brain growth and connectivity have provided potential for better tailoring and evaluation of programs intended to promote healthy child development [2–4]. Pioneering, longitudinal research demonstrates that the early postnatal years are marked by dynamic brain development in humans [5–9]. This descriptive work increased our understanding of brain-behavior connections by revealing differences between males and females [10], noting brain-behavior links in individuals with different cognitive abilities [11], and discovering neural differences in individuals with certain genetic disorders [12]. Here, we seek to harness these approaches to increase understanding of how family poverty may influence children’s brain development with the goal of supporting interventions to reduce some of these negative influences of poverty.

Growing up in poverty has broad and enduring effects on children’s development that include poor physical health, high rates of behavioral problems, and low school achievement, yet the mechanisms causing these effects are not well understood [13]. This represents a critical humanitarian and public health problem because more than half of the world’s poorest people are children. UNICEF reports that among the world’s economically advanced countries, the proportion of children (aged 0 to 17 years) living in relative poverty ranges from 4.7% in Iceland to 25.5% in Romania [14]. This same report lists the relative child poverty rates in Canada and the United States at 13.3% and 23.1%, respectively. In the United States, the proportion of younger children (under age 9 years) living in low income families is even higher, with 21% living in poor families and an additional 23% living in families with incomes just above the poverty threshold [15]. These percentages represent about 15 million children. Thus, neuroscience-informed tools to aid in the creation and assessment of interventions for children in poverty can be very impactful.

To advance progress in this area, we characterize the developmental trajectories of the four major cortical lobes in typically developing children from infancy through early adulthood and examine how growing up in a low-income household is related to that structural development. Our work is based on the integration of two nationally representative cohorts from the NIH Magnetic Resonance Imaging (MRI) Study of Normal Brain Development. Importantly, these data include (1) longitudinal quantitative MRI measures and (2) an indicator of household poverty. Despite significant advancements in neuroscience research on poverty and brain development, we are not aware of data linking volumetric changes in childhood and adolescence with data obtained during infancy. Across middle childhood and adolescence, studies most often rely on quadratic functions that reveal non-linear dynamics of brain development [16]. But the growth of the infant brain is particularly rapid, nearly doubling in size in the first year of life [8]. By utilizing linear mixed models with flexible fractional polynomial parameterization we are able to overcome the statistical challenges inherent in capturing the shape and pace of development over a wide age range. Next, we move beyond modelling average cortical volume to construct sex-specific volume-by-age percentile plots that reveal the considerable variability in regional volumes and structural change, even among heathy, typically developing children. Finally, we test for differences in gray matter development among children growing up in or near poverty, as contrasted to children growing up in middle and higher income families. Our models allow associations between family income and cortical volume to vary as a function of age. As a result, we are able to examine how signficant structural differences detected during childhood may evolve throughout adolescence.

### Participants

Data are derived from the NIH MRI Study of Normal Brain Development, a normative reference database with longitudinal neuroimaging data for a demographically representative sample of infants, children, and adolescents. Data and supporting documentation are available to qualified researchers on the NIMH Data Archive (https://nda.nih.gov/edit_collection.html?id=1151). Details related to study objectives, population-based sampling, subject recruitment, screening procedures, and sample representativeness have been published elsewhere [17–19]. In brief, healthy, typically developing children were recruited at six pediatric study centers (PSCs) across the United States: Children’s Hospital, Boston; Children’s Hospital Medical Center of Cincinnati; Children’s Hospital of Philadelphia; the University of California at Los Angeles; the University of Texas Health Science Center at Houston; and Washington University Saint Louis. Recruitment catchment areas were defined to include ZIP codes within a 30 to 60 mile radius (depending on site) of the PSC. A population-based sampling plan was employed to recruit a demographically representative healthy sample. National demographic data from the U.S. Census Bureau (Census 2000) were used to define enrollment targets based on family income level and race/ethnicity. Targets within the predefined income-by-race/ethnicity demographic cells were distributed across subject age (according to target age distribution) and sex (equal numbers of males and females were targeted for each cell). Region-specific enrollment targets reflecting local population demographics were established for each PSC. Recruitment was monitored continuously by a Clinical Coordinating Center (CCC) to ensure the final sample (enrolled across all PSCs) approximated national demographics. Participating families completed a comprehensive pre-enrollment screening interview. Factors suspected to (1) adversely affect healthy brain development or (2) prevent successful completion of study protocols (e.g. contraindications to MRI) were exclusionary.

The study was organized around two coordinated protocols (`objectives’) reflecting differences in targeted ages at recruitment. The first (OBJ-1) enrolled 431 children and adolescents (207 male, 224 female) from 4 years, 6 months to 18 years across six PSCs. Neuroimaging measures were collected at two-year intervals for a maximum of 3 time points. The second (OBJ-2) enrolled 123 infants and toddlers (69 male, 54 female) from birth to 4 years, 5 months across two of the six PSCs. Neuroimaging measures were collected over a period of 7 years. Intervals between repeated scans ranged from 3 months for the youngest subjects to 1 year, depending on the age of the participant.

The combined sample included 1,057 scans from 486 children with non-missing neuroimaging data (Table 1). Subject age ranged from 5 months to 20 years, 11 months with an equal sample distribution across sex. The sample was racially, ethnically, and economically diverse. Household income ranged from well below to more than eight times the federal poverty level (FPL). Around one-quarter of the sample is classified as poor or near poor (household income below 200% FPL).

**Table 1:** NIH MRI study of normal Brain Development: OBJ-1 and OBJ-2 protocols.

	Mean (%)	SD	Min	Max
Birth weight (grams)^a^	3541.2	472.0	2438.1	5159.6
Age (months)	121.8	66.5	5	251
Age (years)	10.1	5.54	0.42	20.9
Male	48.1%			
Hispanic	11.3%			
Black	10.2%			
White	73.7%			
Household Income^c^				
Below 200% FPL	26.0%			
200 to 400% FPL	43.4%			
Above 400% FPL	30.6%			
Missing	2.6%			
**OBJ-1** (N = 399, n = 831)	Mean (%)	SD	Min	Max
Age (years)	12.4	3.9	4.9	20.9
Age at first scan (years)^b^	11.1	3.6	4.9	20.3
Number of scans/subject:	2.1	0.73	1	3
1	23.1%			
2	45.6%			
3	31.1%			
Male	46.7%			
Household Income				
Below 200% FPL	24.8%			
200 to 400% FPL	43.6%			
Above 400% FPL	28.9%			
Missing	2.7%			
**OBJ-2** (N = 87, n = 226)	Mean (%)	SD	Min	Max
Age (months)	22.4	15.5	5	102
Age at first scan (months)^b^	15.9	11.1	5	54
Number of scans/subject:	2.6	1.6	1	7
1	28.7%			
2	31.0%			
3	19.5%			
4+	20.8%			
Male	53.1%			
Household Income				
Below 200% FPL	27.4%			
200 to 400% FPL	37.3%			
Above 400% FPL	33.1%			
Missing	2.2%			

Analysis sample includes 1,057 observations from 486 unique children with non-missing neuroimaging data. OBJ-1 protocol enrolled children 4.5 to 18 years. OBJ-2 protocol enrolled newborns to children 4.5 years.

^a^ We do not observe birthweight for three in-sample children. One child with very low birth weight (< 1500 grams) was excluded.

^b^ Refers to first successfully segmented scan.

^c^ Nineteen children with missing data on family income and/or family size are excluded from poverty analyses.

Written informed consent was obtained for all subjects and/or their legal guardians at each study time point. All protocols and procedures were approved by the relevant Institutional Review Board at each PSC (Children’s Hospital, Boston; Children’s Hospital Medical Center of Cincinnati; Children’s Hospital of Philadelphia; University of Texas Health Science Center at Houston; Washington University St. Louis; and University of California, Los Angeles). All procedures were in accordance with the Helsinki Declaration.

### Image acquisition and tissue segmentation

T1- and T2-weighted anatomical scans were obtained with a GE (General Electric, Milwaukee, WI) or Siemens (Siemens AG, Erlangen, Germany) MRI scanner for all participants. MR acquisition and processing for this study have been extensively detailed in past reports [17, 18]. In brief, for child and adolescent participants (OBJ-1), quality control checks were completed and then a mutual information-based registration procedure was completed to normalize all images into Montreal Neurological Institute (MNI) stereotaxic space. T1 and T2 volumes were then mapped non-linearly to this template, non-brain tissue (e.g., skull; dura) was masked, and then tissue type was identified using custom tissue segmentations based on the age- and gender-composition of our sample. Anatomical labels were then mapped onto MR volumes, specifically 4 lobular gray matter volumes (GM) and total white matter (WM) across the brain. For infant MR data (OBJ-2), quality control checks were completed and all structural scans (T1 and T2 volumes) were masked for non-brain tissue, and then bias corrected with nonparametric non-uniform intensity normalization methods to reduce the impact of intensity inhomogeneity [20–22]. All images were segmented via an Expectation-Maximization (EM) algorithm with infant brain atlases representing subject-independent population information [23]. This segmentation involved two iterative steps: 1) a registration step for aligning an age-specific atlas onto a given image and 2) a segmentation step for estimating brain tissues using the MRI intensity distribution from the image in conjunction with the aligned tissue probability maps from atlas. A brain atlas labeled with gray matter, white matter, and the four primary lobes of the brain (i.e., frontal, temporal, parietal, occipital) was employed to label the whole-brain. This atlas was originally defined on the Montreal Neurological Institute (MNI) single subject brain MR image and was later adapted for infant neuroimages [23]. For objective 1, T1-weighted volumes were collected with a native resolution of 1–1.5mm, while T2-weighted volumes were acquired at 2mm. For objective 2, T1-and T2- weighted volumes were aquired with a slice thicknesses of 3mm. Supplemental analyses were undertaken to see if protocol variation, i.e. differences in acquisition slice thickness, would preclude quantitative comparisons across the objectives. Aquistion parameters were found to have little to no effect on volumetric quantification, at least for large lobular parcels (S1–S5 Figs).

### Modeling brain development

To assess structural brain development, we model growth trajectories for each cortical lobe in healthy males and females from age 5 months to 20 years, 11 months. Three-level linear mixed models (LMM) combine cross-sectional and longitudinal data and take into account the hierarchical nature of the data: over 1,000 structural MRI scans (level 1) acquired longitudinally for 487 unique subjects (level 2) across 6 study centers (level 3). Lobe size, i.e. gray matter volume, acquired for subject *i* during follow up *t* at study center *C* is a function of subject sex and age:

$${\mathrm{volume}}_{iCt}=f({\mathrm{sex}}_{i},{\mathrm{age}}_{it})+v_{i}+u_{iC}+\in_{iCt}$$ (1)

The fixed portion of the LMM, *f*(*sex_i_, age_it_*), is a smooth function consisting of two transformations of subject age. To accommodate anticipated sexual dimorphism, the fixed effects structure is fully interacted by sex, allowing sex differences in average GM volume and GM volume growth. Models include random intercepts at both the study center (*u_iC_*) and individual-within-center (*v_i_*) levels. Robust standard errors allow for heteroscedasticity with respect to study center. Additional random effects were tested and subsequently rejected.

For each lobe we select a best-fit second-order fractional polynomial (FP) for the fixed effects of age on lobe GM volume. The conventional polynomial, i.e. quadratic, trajectory is not imposed, but subsumed in the set of candidate FP models. This approach offers the greater flexibility in curve shape required to adequately capture brain dynamics from infancy through adulthood. Candidate models are compared using the log likelihood (-2LL), Akaike’s information criterion (AIC), and Schwarz’s Bayesian information criterion (BIC). Comprehensive treatment of modelling growth curves with FPs is provided by others [24]. Details related to the selection of best-fit models are available in a statistical appendix (S1 File).

Taking the first derivative with respect to age, yields estimates of regional gray matter growth (measured in mm^3^/month):

$${\mathrm{growth}}_{iSt}=\frac{\partial }{\partial age_{iSt}}f({\mathrm{sex}}_{i},{\mathrm{age}}_{iSt})$$ (2)

### Volume-for-age percentile plots

Beyond estimating average gray matter volume and growth, we construct sex-specific volume-for-age percentile plots following recent procedures for modeling growth trajectories and rates of change [8]. These growth tables allow one to assess the statistical distribution of normal gray matter development in the frontal, temporal, parietal and occipital lobes. Using best-fit gray matter trajectories in Eq (1), we calculate the difference between subject gray matter volume (volume_iCt_) and the expected or average volume for a child of the same sex and age. We then model the square of these residuals as a smooth, first-order function of age. The described models yield sex-specific estimates of a population standard deviation for gray matter volumes in regions of interest, that are allowed to vary with age. Volume for a specified age-sex-percentile is obtained according to the inverse of the associated normal cumulative distribution function.

### Children from low income families

Total family income is reported in ranges: $0-$5,000; $5,001-$10,000; $10,001-$15,000; $15,001-$25,000; $25,001-$35,000; $35,001-$50,000; $50,001-$75,000; $75,001-$100,000; and $100,001-$150,000. We adjust family income, measured at the categorical midpoint, according to federal poverty guidelines. Around one-quarter of the sample is classified as poor or near poor, with adjusted total family income falling below 200% of the federal poverty level (FPL). Researchers and policy advocates commonly use household income at or below 200% FPL as an indicator of economic security. Income distributions for the OBJ-1 and OBJ-2 cohorts are similar (Table 1). Reported income is overwhelmingly stable during the sample period, with very few families transitioning into or out of poverty.

Prior research has shown that the relationship between income and brain structure is not continuous but, rather, strongest among children in low-income households [25, 26]. To test for potential differences in structural brain development among children growing up in or near poverty, we re-estimate the best-fit GM trajectories in Eq (1) for the frontal, temporal, parietal and occipital lobes. Augmented models include an indicator of low income status (family income below 200% FPL) and associated interactions with the two transformations of subject age. Alternative specifications also adjust for birth weight, an indicator of both early health status and initial head size. We also consider the sensitivity of our estimates to the selection of alternative income thresholds, e.g., family income below the federal poverty level. Effects of income are estimated through non-parametric bootstrapping, based on 1,000 draws with replacement.

## Results

### Typical brain growth

**Fig 1:**
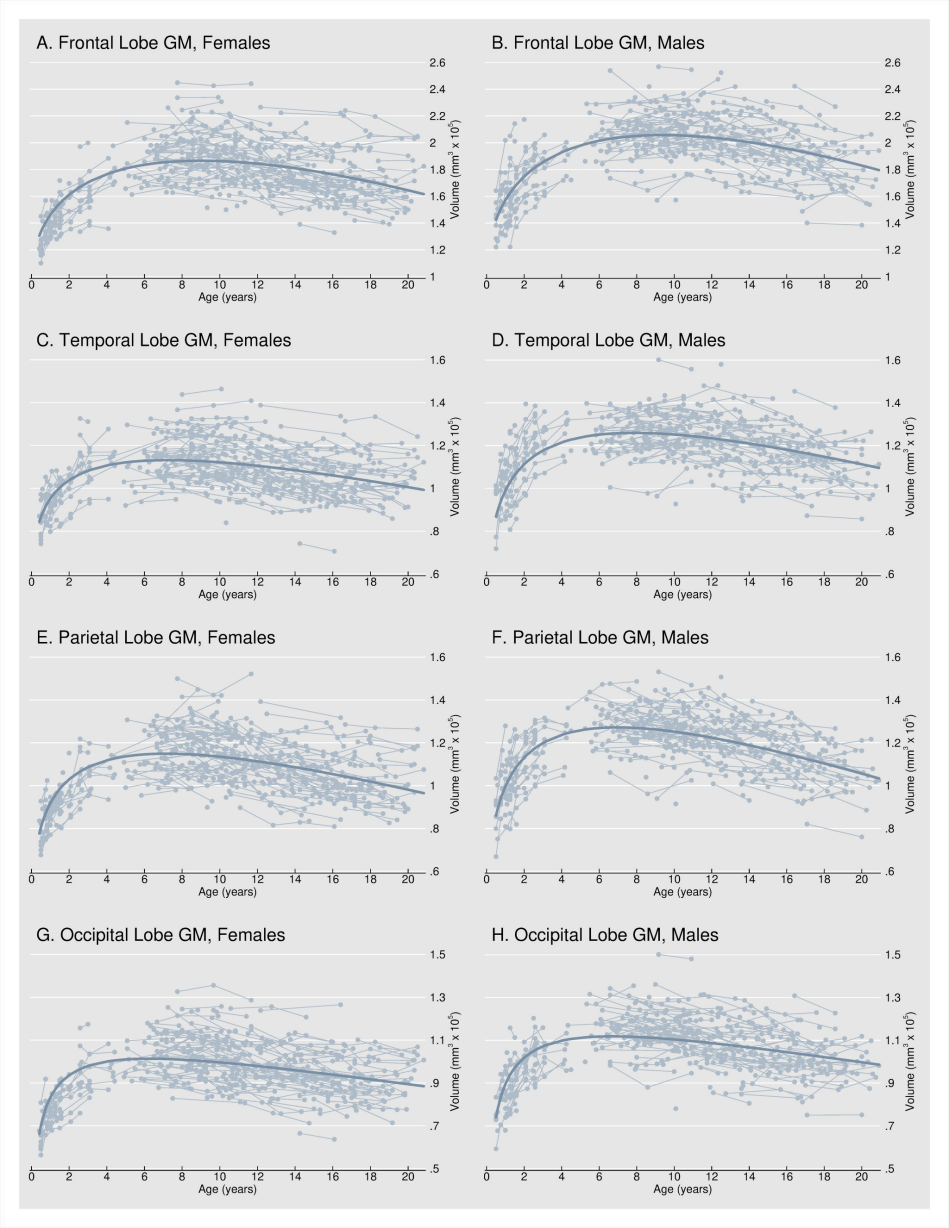
Regional gray matter volume trajectories. Fig 1 shows modeled GM volume trajectories for the frontal, temporal, parietal, and occipital lobes. Estimated trajectories outline typical GM development from infancy through young adulthood in males and females. Spaghetti plots trace individual subject’s observed dynamics.

Fig 1 shows modeled GM volume trajectories for the frontal, temporal, parietal, and occipital lobes. Estimated trajectories based on Eq (1) outline typical, i.e., average, GM development from infancy through young adulthood in males and females. Spaghetti plots trace individual subject’s observed dynamics. Consistent with previous reports from post-infancy cohorts, we observed that cortical gray matter volumes generally follow an inverted U-shaped developmental trajectory [10, 16]. Cortical gray matter volumes increased during infancy and early childhood, peaked in late childhood, and then decreased throughout adolescence and early adulthood. Beyond a similar U-shaped path, developmental trajectories of the four major cerebral lobes are distinct. GM volume peaks earliest in the occipital lobe (around 6 years of age) and latest in the frontal lobe (around 9 years of age). These observations are, again, consistent with prior reports that GM initially peaks in primary sensorimotor areas and that maturation of higher order areas that integrate functions, such as the dorsolateral prefrontal cortex, inferior parietal and superior temporal gyrus occurs later in development [2]. While early studies report that cortical GM volumes peak around the onset of puberty [10, 16], our findings that gray matter volumes are highest between 6 to 10 years of age are in line with other recent longitudinal studies [3, 27, 28].

**Fig 2:**
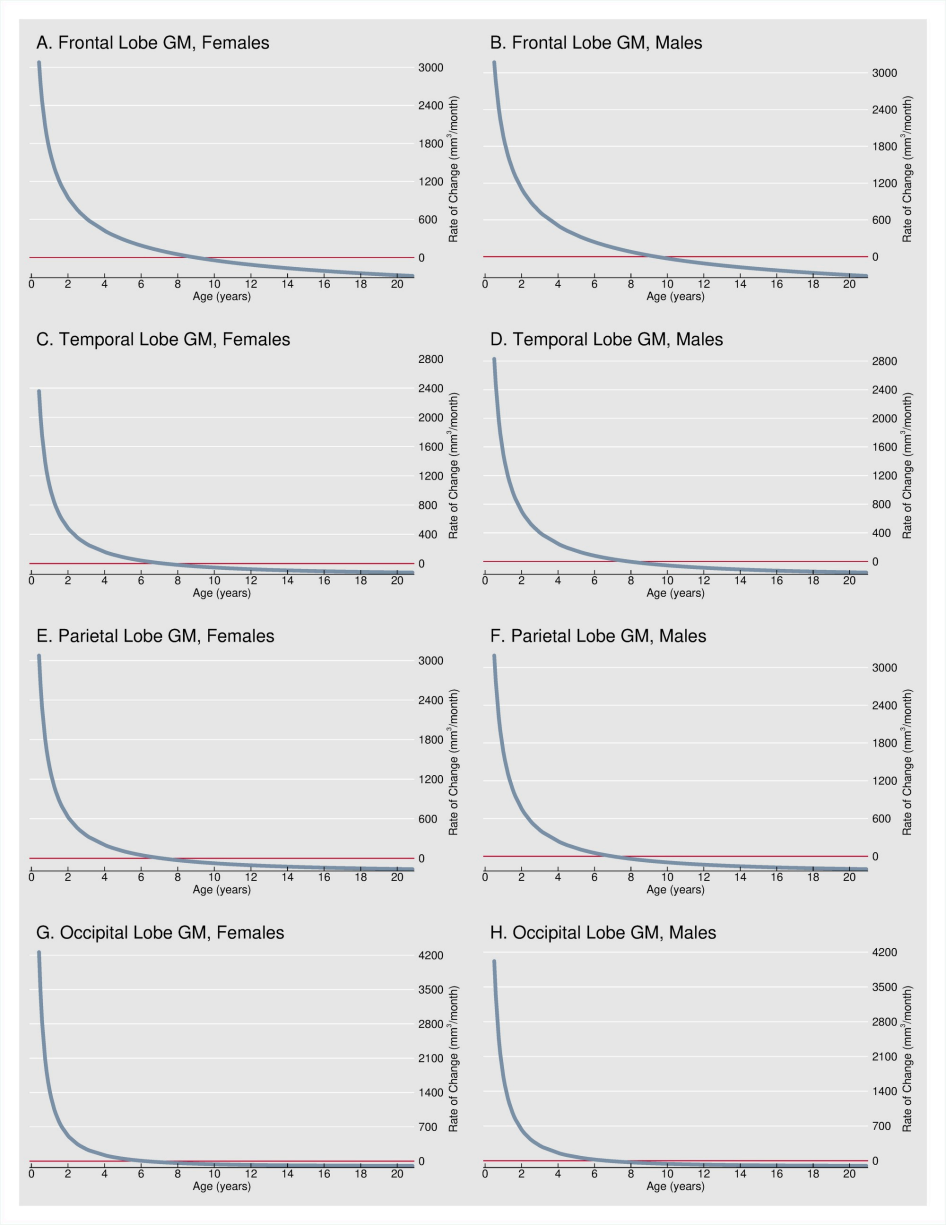
Monthly growth rate. Fig 2 shows the estimated rate of growth expressed as mm^3^/month for GM in the frontal, temporal, parietal, and occipital lobes. Relative growth rates expressed as a percentage of the predicted lobe size at the same age (%/month) were examined in supplementary analyses (S6 Fig).

Fig 2 shows the estimated rate of growth expressed as mm^3^/month for GM in the frontal, temporal, parietal, and occipital lobes. Patterns are qualitatively similar if, instead, we express the monthly growth rate as a percentage of predicted lobe size at the same age (S6 Fig). GM volume increases most rapidly shortly after birth, increases at a decreasing rate throughout infancy and adolescence, and decreases throughout adolescence and into young adulthood. Near age 5 months–the youngest age observed in our data–gray matter had a rate of growth of 2.1%/month (frontal lobe) to 3.7%/month (parietal lobe) increases in regional volume. In contrast, by age 4–5 years, growth rates (as a percentage of regional volumes) have already decreased by a factor of 10 or more.

**Fig 3:**
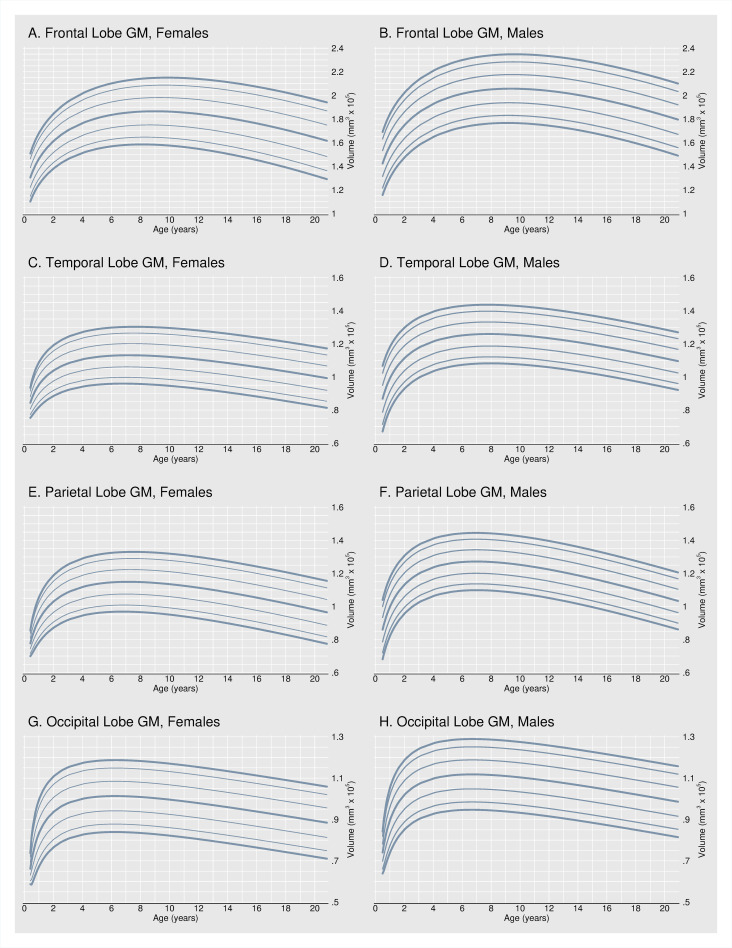
Volume-for-age percentiles. Fig 3 shows volume-for-age percentile plots for GM volume in the frontal, temporal, parietal, and occipital lobes. Charts include the 5th (bolded), 10th, 25th, 50th (bolded), 75th, 90th, and 95th (bolded) percentile curves.

Fig 3 shows volume-for-age percentile plots, similar to clinical growth charts, for GM volume in the frontal, temporal, parietal, and occipital lobes. Beyond summarizing a typical or average developmental trajectory, volume-for-age percentile plots describe the statistical distribution of healthy GM development for females and males. Charts include the 5th, 10th, 25th, 50th, 75th, 90th, and 95th percentile curves. Even in a sample of healthy, typically developing children, we find that the size of the four major cerebral lobes is highly variable. Holding age constant, lobular GM volume can range by as much as a factor of 2.

### Differences in brain growth by sex

Longitudinal MRI studies in child and adolescent cohorts consistently report larger volumes in males as compared to females [29–32]. Consistent with this prior work, we find that, on average, male brains are 10% larger consistent across regions of interest and stable over much of the lifespan. These sex differences are apparent in infancy and stabilize by age 3. Yet, sex differences in trajectories are less clear. Therefore we examined the effect of sex on the overall shape of developmental trajectories, testing for differences in both the timing of peak gray matter volume and rates of volumetric change (growth). All sex differences were bootstrapped; 95% bias-corrected confidence intervals are based on 1,000 replications.

The magnitude of sex differences in rates of growth varied across regions of interest and over the course of development. Analyses of unadjusted growth rates (mm^3^/month) suggest that gray matter volume increases more rapidly in males compared to females during infancy and early childhood. The relatively rapid growth rate among males persists over the first 3 (parietal lobe) to 7 (frontal lobe, temporal lobe) years of life. In the case of the parietal lobe, statistically significant sex differences were detected during adolescence and early adulthood; GM volumes decreased more rapidly in males compared to females between ages 11 and 20 years. In general, observed male-female differences in gray matter growth rates are not robust to adjustments for region size. The temporal lobe is one exception; statistically significant differences in gray matter growth from age 5 months to 7 years are consistent across comparisons of raw and adjusted rates of change. While model predictions suggest GM volumes (in the frontal, temporal and occipital lobes) reach a maximum 5 to 7 months earlier in females compared to males, we detected no statistically significant sex differences in peak timing.

### Differences in brain growth by family income

**Fig 4:**
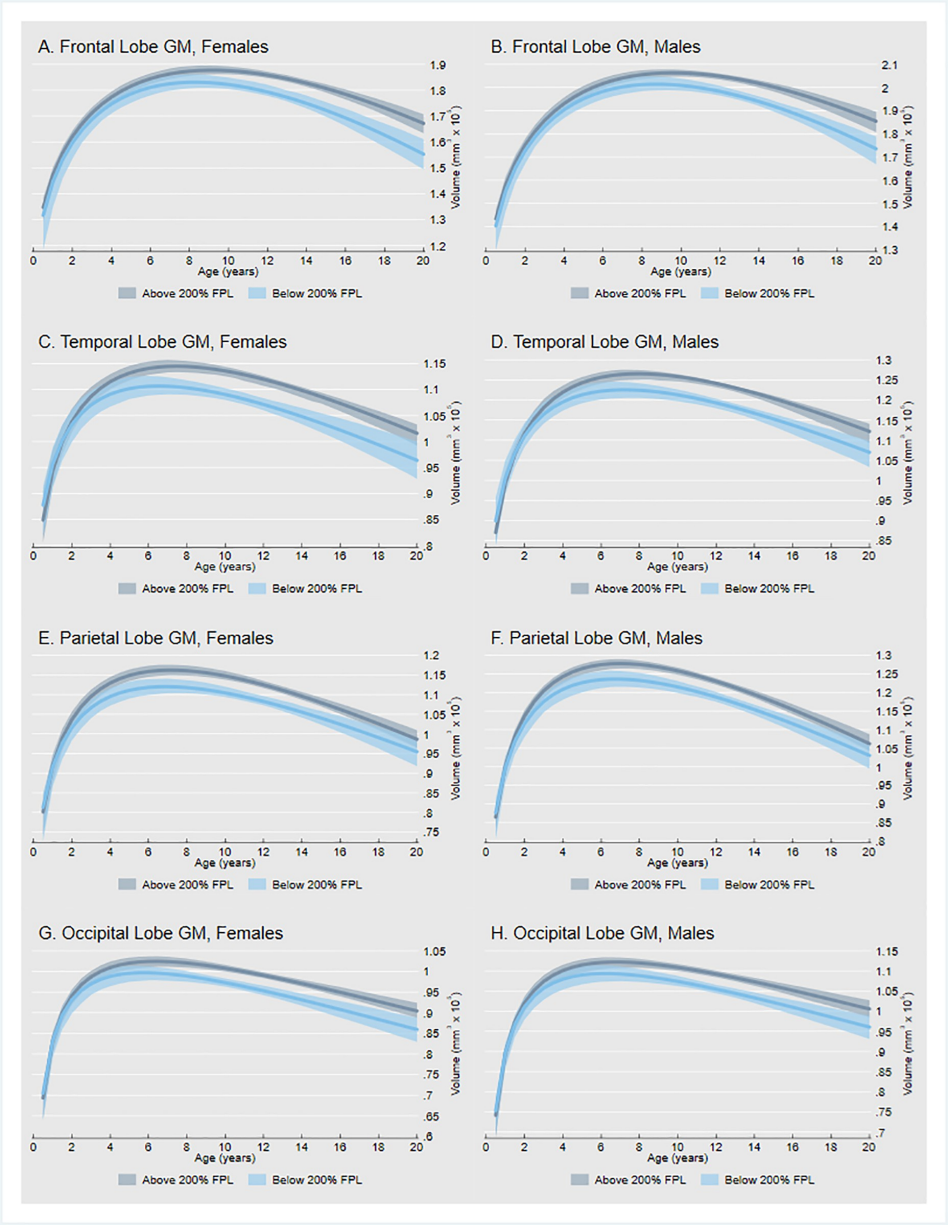
Regional gray matter volume trajectories by family income. Fig 4 shows modeled GM volume trajectories for the frontal, temporal, parietal, and occipital lobes by household income. Gray lines outline typical GM development in children from nonpoor families with household income above 200% of the federal poverty level (FPL). Blue lines outline GM development in children from poor and near poor families with household income below 200% of the FPL.

We find that children from poor and near poor families have lower average GM volumes in the frontal, temporal, parietal, and occipital lobes (Fig 4). The magnitude of income differences in GM volume varied across regions of interest and over the course of development. All income differences were bootstrapped; 95% bias-corrected confidence intervals are based on 1,000 replications. Our findings are robust to the inclusion of a control for birth weight, an indicator of both early health status and initial head size (S7 Fig). We did not have sufficient power to test whether the effects of low income vary by sex. If we restrict our focus to children from the poorest households, we observe larger GM differences earlier in childhood, though the confidence intervals are wider (S8 Fig).

#### Frontal lobe

Although neonates have similar frontal GM volumes, statistically significant income differences were detected by age 6.5 years (β = -3574.6; 95% CI: -6673.0, -74.6) and found to increase steadily through late childhood and adolescence. By age 20, estimated frontal GM volumes are 6.4% (males) to 7.1% (females) smaller in children living in or near poverty.

#### Temporal lobe

Statistically significant income differences in temporal GM volumes were detected earlier in childhood, by age 4 years (β = -2406.8; 95% CI: -4942.7, -183.8). The income gap was found to increase in magnitude from childhood through adolescence. By age 20, estimated temporal GM volumes are 4.7% (males) to 5.1% (females) smaller in children living in or near poverty.

#### Parietal lobe

Income disparities in the parietal lobe are apparent at very young ages. Statistically significant income differences in parietal GM volumes were detected by age 2.5 years (β = -2489.7; 95% CI: -5981.2, -314.4). The gap between poor and non-poor children was found to peak around age 11 years before narrowing slightly. At age 20, estimated parietal GM volumes are 3.0% (males) to 3.2% (females) smaller in children living in or near poverty.

#### Occipital lobe

In the case of the occipital GM volumes, statistically significant income differences were detected by age 4 years (β = -2114.3; 95% CI: -4454.3, -90.9). Once established, the gap increased gradually over the age span studied. By age 20, estimated occipital GM volumes are 4.5% (males) to 5.0% (females) smaller in children living in or near poverty.

## Discussion

It has been difficult to formulate a comprehensive picture of foundational neurodevelopment that extends from birth through adulthood. Here, we leverage advances in statistical modeling, namely linear mixed models with fractional polynomial parameterization, to overcome the statistical challenges inherent in capturing the shape and pace of development over the first 20 years of life. This novel approach provides a framework to better understand structural brain development across infancy, childhood, and adolescence.

Our findings confirm two general conclusions about typical, or average, brain development. First, consistent with patterns observed by others [2], we found lobular development in sensorimotor portions of the brain peaked earlier than higher-order areas of the cortex. Also consistent with more recent work [3], we found gray matter volumes peaked in childhood (prior to the onset of puberty) and then decreased over the course of adolescence. Our results, in line with previous reports [8], capture the significant and rapid changes occuring in the brain in the months soon after birth.

Beyond simply modelling average or typical gray matter development, the present data describes sex-specific volume-for-age percentile plots. These plots allow assessment of the *statistical distribution* of normal gray matter development. Indeed, our results underscore the considerable variability in the structural development of the cortical lobes–even among healthy, typically developing children. Future research should aim to add the specificity needed for practical or clinical applications tailored to individuals.

We used our models to test for differences in gray matter development among children growing up in or near poverty, as compared to children growing up in middle- and higher-income families (similar studies could be conducted to test for differences by premature birth, exposure to toxins, maltreatment, or other experiences). While earlier studies linking socioeconomic disandvantage to neuroanatomical differences estimate an average income gap, e.g., throughout childhood and adolescence [25], our models allow associations between family income and cortical volumes to vary as a function of age [26, 33]. Although associations between SES and brain development have been studied in infancy [34] and in childhood and adolescence [25, 26, 33, 35], these literatures exist in isolation. No studies, to our knowledge, have examined how significant structural differences detected in the first few postnatal years evolve throughout adolescence. We found differences in gray matter in the temporal, parietal, and occipital lobes between poor and nonpoor children were present by 2.5–4 years of age, and these gaps remained throughout the ages we studied–through 20 years. Differences in gray matter in the frontal lobe appeared later in development (ages 6.5 years) but continued to increase over the ages we studied.

### Limitations

It is not trivial to collect a national, demographically representative sample of brain growth with repeated measures on individual children. To do so required that some compromises were necessary to overcome many pragmatic challenges. Although we use the best currently available data, it is still limited in both the total number of observations and the number of observations at each age. In particular, there were few observations of ages near the end point of Objective 2 (age 4 years, 5 months) and the beginning of Objective 1 (age 4 years, 6 months). Yet, the entirety of the data set still maintains the power of two connected panel data sets that covers development from infancy. Newer neuroimaging data sets bring other advantages, but often do not begin in early infancy, do not contain repeated measures on individual children, or do not include measures of household income.

A second issue is that the scans were conducted at six different sites across the United States, using two different types of scanners. We do control for site in our analyses, but these differences no doubt add variance to the data. At the same time, an argument can be made that any finding about normative brain growth that is truly robust should persist across MRI hardware. On this view, the variability in scanners might lend more confidence about these observed growth curves.

A third and important issue is that there were some differences in parameters for the Objective 1 and Objective 2 scans. One difference was in slice thickness across child/adolescent and infant scans. Relatedly, native resolution of 3mm^2^, by current standards, does not afford a very precise segmentation, particularly for infants. This would give cause for concern if we were attempting to localize small or difficult to image structures in the brain. In relatively large regions of the brain, however, supplemental analyses indicated near perfect correlation in lobular volumes derived from 3mm^2^ versus 1mm^2^ scan resolution (S5 Fig). Moving forward, pending availability of high-resolution structural MRI in a large, longitudinal study of infants and children, our approach to modelling structural brain development (i.e., using flexible fractional polynomial specifications to connect the significant neural changes occurring just after birth to the subtler refinements in the brain occurring in childhood and adolescence) could be applied to subcortical structures and other anatomically defined regions of the brain that have been linked to specific domains of cognitive and behavioral functioning.

There is growing recognition of the need to incorporate population science approaches, including purposive sampling, in neuroscience research [36, 37]. In order to minimize biases that can be present in samples of convenience and enhance the generalizability of findings, the NIH MRI Study of Normal Brain Development employed a population-based sampling plan that was designed to reflect regional as well as national distributions of family income level and race/ethnicity. Even so, children residing in rural locations or in families with lower levels of parental educational attainment may be underrepresented. Further, results from our models only generalize to typically developing children, the target population of the NIH MRI Study of Normal Brain Development.

Finally, the reported links between family income and brain structure are correlational. It is possible that differences in GM trajectories observed in poor and near poor children could be caused by a third factor that is tied both to family income and brain structure. The NIH MRI Study of Normal Brain Development was designed to characterize healthy brain development in typically developing children. The original study’s detailed screening procedures and strict exclusionary criteria help us to rule out a number of potentially confounding factors, including significant physical or behavioral conditions of the child, a family history of inherited neurological or psychiatric disorders, and intrauterine exposures to substances (e.g., illicit drugs, tobacco, alcohol, or certain medications) suspected to alter brain structure or function. They also help to mitigate the potential for adverse selection of sample families based on unobserved factors (e.g., families who may volunteer to participate in the study out of concern for a child’s health or developmental progress). While it is possible that income-related differences in GM trajectories reflect, in part, heritable influences, studies employing genotyping methods show that associations between socioeconomic status and differences in brain structure persist independently of genetic ancestry [26, 38].

## Conclusion

This study presents GM trajectories based upon a demographically representative sample of infants, children and adolescents. Volume-for-age percentile plots reveal the considerable variability in structural brain development among healthy, typically developing children. Across the four lobes studied, however, we found systematic differences in GM volumes by family income. These income differences were observed for both males and females. With replication and further empirical refinement, these charts, like those long established for weight and height, could serve as one potential tool to more precisely monitor or track neurodevelopment. Continued refinement of our understanding of the development of the human brain holds tremendous promise for earlier detection of neurodevelopmental differences, determination of developmental periods where interventions may be most effective, and evaluation of policies aimed at supporting optimal child development.

## Supporting information

S1 Fig Frontal lobe GM volume, NIH MRI study of Normal Brain Development OBJ-1 and OBJ-2 cohorts. As a first step in assessing the feasibility of combining the OBJ-1 and OBJ-2 cohorts of the NIH MRI Study of Normal Brain Devolpment, we visually inspected a spaghetti plot that traced individual trajectories of GM volume in the frontal lobe. Congruence in observed GM volumes across the two cohorts, particularly between ages 4 and 6 years, supports efforts to chart brain development from infancy through adolescence.
(TIF)

S2 Fig Temporal lobe GM volume, NIH MRI study of Normal Brain Development OBJ-1 and OBJ-2 cohorts. As a first step in assessing the feasibility of combining the OBJ-1 and OBJ-2 cohorts of the NIH MRI Study of Normal Brain Devolpment, we visually inspected a spaghetti plot that traced individual trajectories of GM volume in the temporal lobe. Congruence in observed GM volumes across the two cohorts, particularly between ages 4 and 6 years, supports efforts to chart brain development from infancy through adolescence.
(TIF)

S3 Fig Parietal lobe GM volume, NIH MRI study of Normal Brain Development OBJ-1 and OBJ-2 cohorts. As a first step in assessing the feasibility of combining the OBJ-1 and OBJ-2 cohorts of the NIH MRI Study of Normal Brain Devolpment, we visually inspected a spaghetti plot that traced individual trajectories of GM volume in the parietal lobe. Congruence in observed GM volumes across the two cohorts, particularly between ages 4 and 6 years, supports efforts to chart brain development from infancy through adolescence.
(TIF)

S4 Fig Occipital lobe GM volume, NIH MRI study of Normal Brain Development OBJ-1 and OBJ-2 cohorts. As a first step in assessing the feasibility of combining the OBJ-1 and OBJ-2 cohorts of the NIH MRI Study of Normal Brain Devolpment, we visually inspected a spaghetti plot that traced individual trajectories of GM volume in the occipital lobe. Congruence in observed GM volumes across the two cohorts, particularly between ages 4 and 6 years, supports efforts to chart brain development from infancy through adolescence.
(TIF)

S5 Fig Comparison of volumetric measures across the OBJ-1 and OBJ-2 cohorts of the NIH MRI study of Normal Brain Development. Supplemental analyses were undertaken to determine whether differences in MR acquisition slice thickness would preclude quantitative comparisons across the OBJ-1 and OBJ-2 cohorts. All scans for a subsample (n = 20) of OBJ-1 participants with 1mm T1-weighted images were resampled to match the typical acquisition parameters used in the OBJ-2 cohort, i.e. 3mm slice thickness. Orginal and recalculated lobular volumes were compared. A high degree of correlation (all r > 0.99) indicates that differences in slice thickness had little to no effect on volumetric quantification, at least for the large lobular parcels considered in this study.
(TIF)

S6 Fig Monthly growth rate. In S6 Fig, we plot the estimated relative growth rate (%/month) for GM in the frontal, temporal, parietal, and occipital lobes. Relative growth rates are expressed as a percentage of the predicted lobe size at the same age.
(TIF)

S7 Fig Regional gray matter volume trajectories by family income. S7 Fig shows modeled GM volume trajectories for the frontal, temporal, parietal, and occipital lobes by household income. Gray lines outline typical GM development in children from nonpoor families with household income above 200% of the federal poverty level (FPL). Blue lines outline GM development in children from poor and near poor families with household income below 200% of the FPL. Models include birth weight, an indicator of both early health status and initial head size, as a covariate.
(TIF)

S8 Fig Regional gray matter volume trajectories by family income. S8 Fig shows modeled GM volume trajectories for the frontal, temporal, parietal, and occipital lobes by household income. Gray lines outline typical GM development in children from nonpoor families with household income above 100% of the federal poverty level (FPL). Blue lines outline GM development in children from poor families with household income below 100% of the FPL.
(TIF)

S1 File Statistical appendix. (DOCX)
