# Supplementary figures and images for: Low household income and neurodevelopment from infancy through adolescence
Source: PLoS One. 2022 Jan 26;17(1):e0262607. doi: 10.1371/journal.pone.0262607 (PMC8791534; doi:10.1371/journal.pone.0262607)

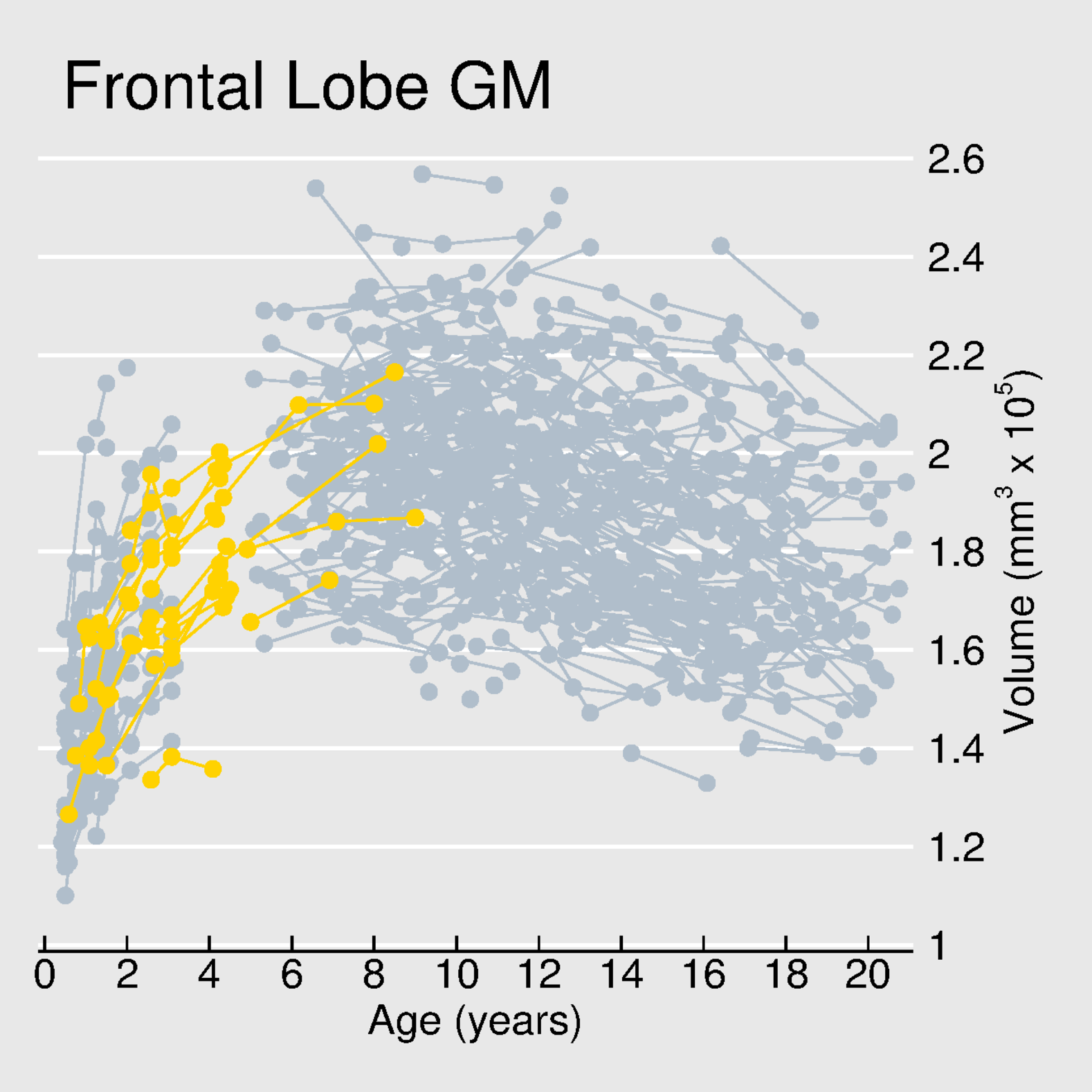

Supplement: S1 Fig — As a first step in assessing the feasibility of combining the OBJ-1 and OBJ-2 cohorts of the NIH MRI Study of Normal Brain Devolpment, we visually inspected a spaghetti plot that traced individual trajectories of GM volume in the frontal lobe. Congruence in observed GM volumes across the two cohorts, particularly between ages 4 and 6 years, supports efforts to chart brain development from infancy through adolescence. (TIF) [file pone.0262607.s001.tif]

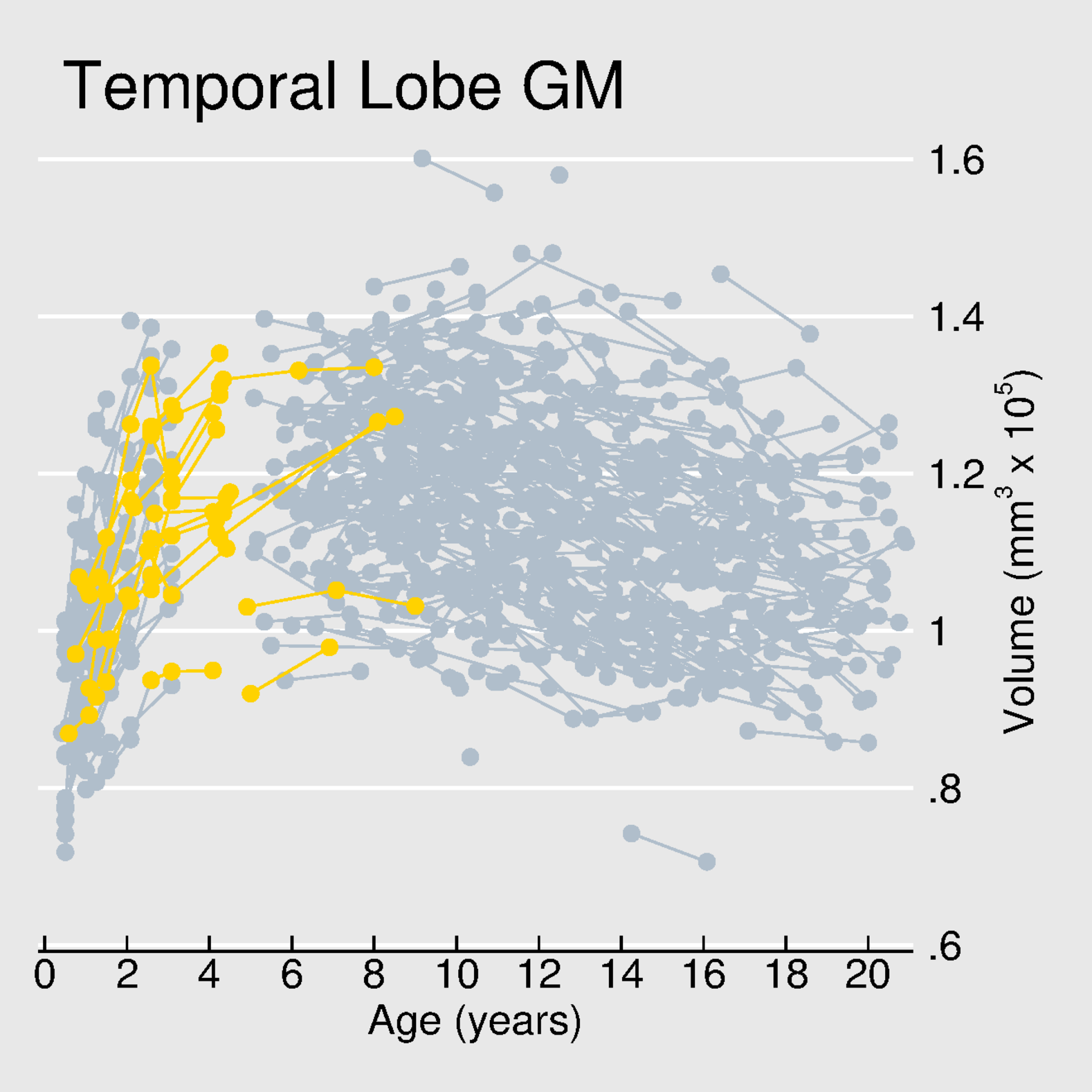

Supplement: S2 Fig — As a first step in assessing the feasibility of combining the OBJ-1 and OBJ-2 cohorts of the NIH MRI Study of Normal Brain Devolpment, we visually inspected a spaghetti plot that traced individual trajectories of GM volume in the temporal lobe. Congruence in observed GM volumes across the two cohorts, particularly between ages 4 and 6 years, supports efforts to chart brain development from infancy through adolescence. (TIF) [file pone.0262607.s002.tif]

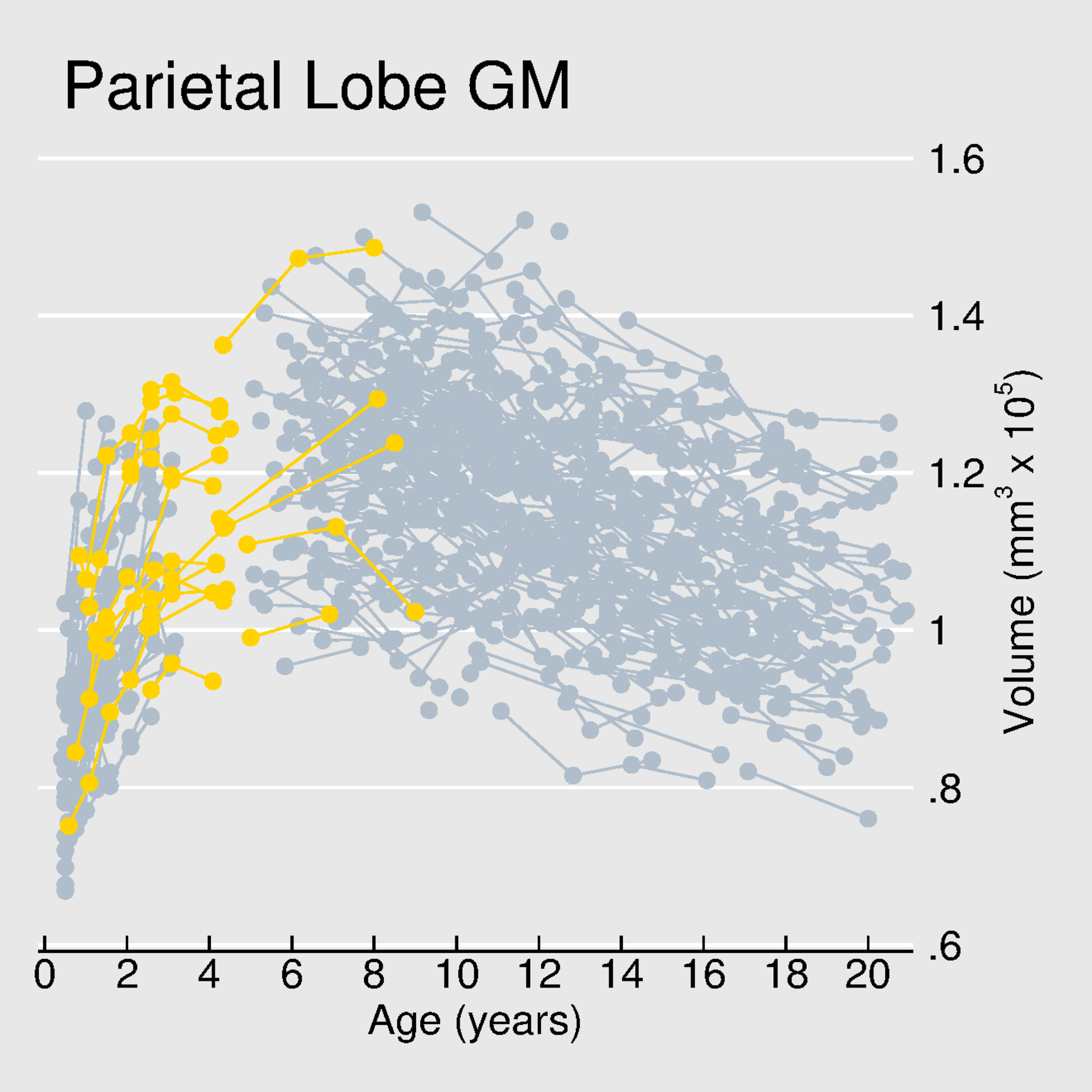

Supplement: S3 Fig — As a first step in assessing the feasibility of combining the OBJ-1 and OBJ-2 cohorts of the NIH MRI Study of Normal Brain Devolpment, we visually inspected a spaghetti plot that traced individual trajectories of GM volume in the parietal lobe. Congruence in observed GM volumes across the two cohorts, particularly between ages 4 and 6 years, supports efforts to chart brain development from infancy through adolescence. (TIF) [file pone.0262607.s003.tif]

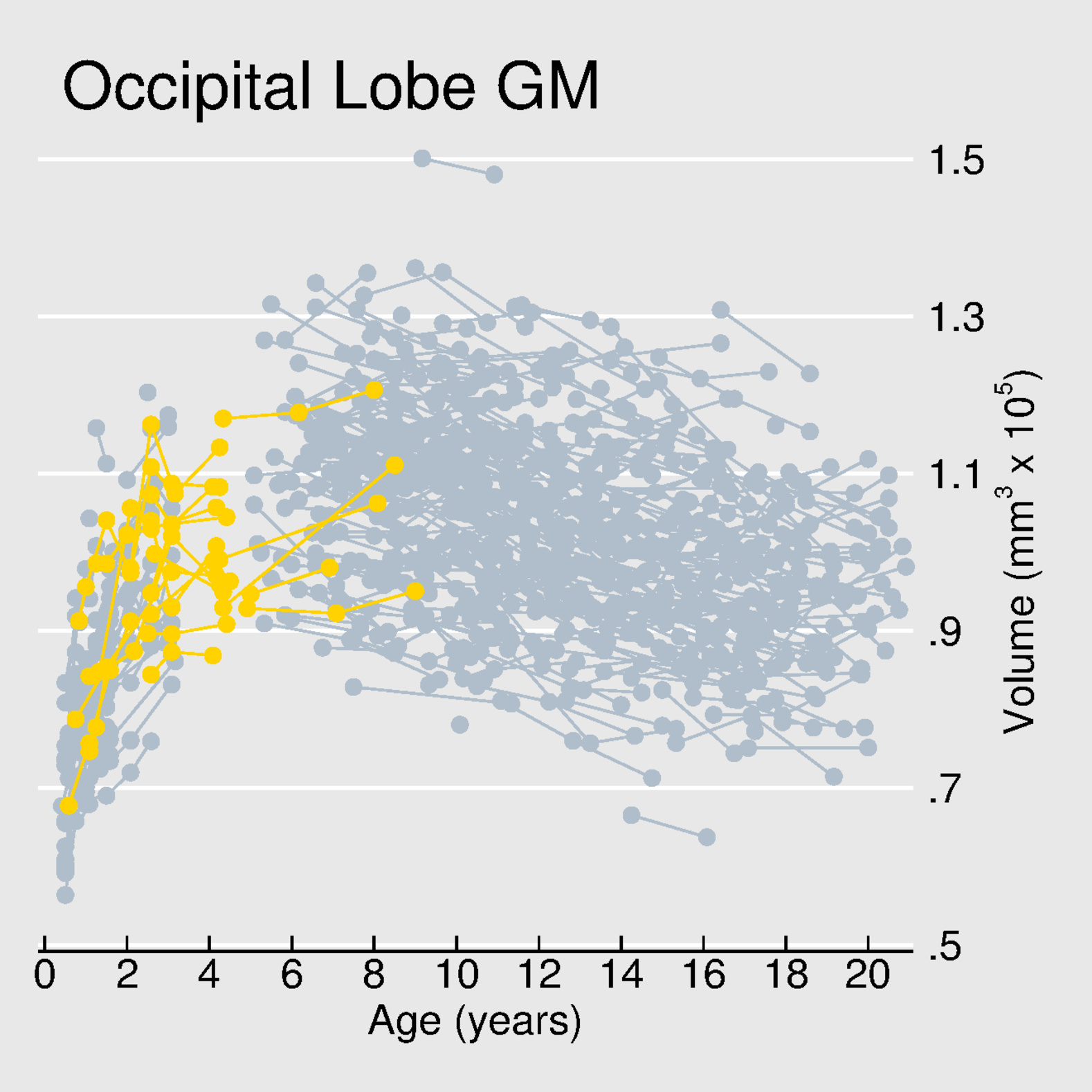

Supplement: S4 Fig — As a first step in assessing the feasibility of combining the OBJ-1 and OBJ-2 cohorts of the NIH MRI Study of Normal Brain Devolpment, we visually inspected a spaghetti plot that traced individual trajectories of GM volume in the occipital lobe. Congruence in observed GM volumes across the two cohorts, particularly between ages 4 and 6 years, supports efforts to chart brain development from infancy through adolescence. (TIF) [file pone.0262607.s004.tif]

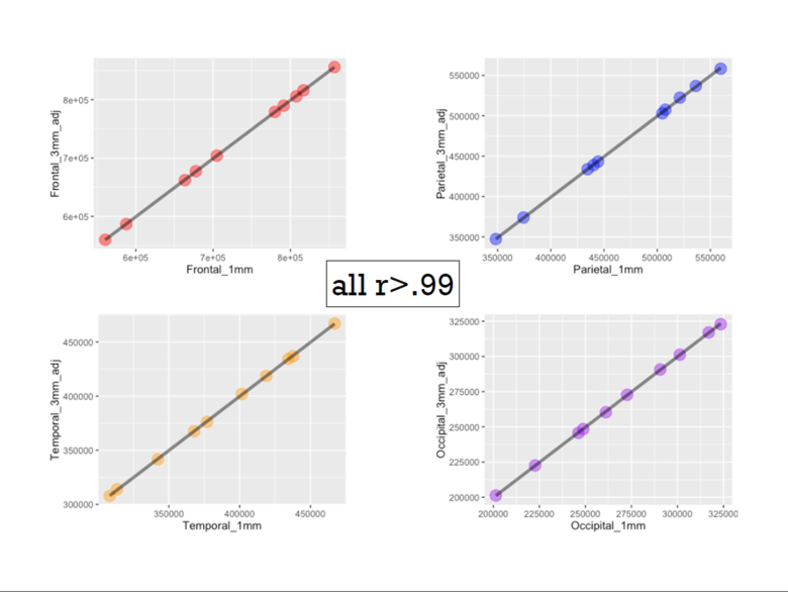

Supplement: S5 Fig — Supplemental analyses were undertaken to determine whether differences in MR acquisition slice thickness would preclude quantitative comparisons across the OBJ-1 and OBJ-2 cohorts. All scans for a subsample (n = 20) of OBJ-1 participants with 1mm T1-weighted images were resampled to match the typical acquisition parameters used in the OBJ-2 cohort, i.e. 3mm slice thickness. Orginal and recalculated lobular volumes were compared. A high degree of correlation (all r > 0.99) indicates that differences in slice thickness had little to no effect on volumetric quantification, at least for the large lobular parcels considered in this study. (TIF) [file pone.0262607.s005.tif]

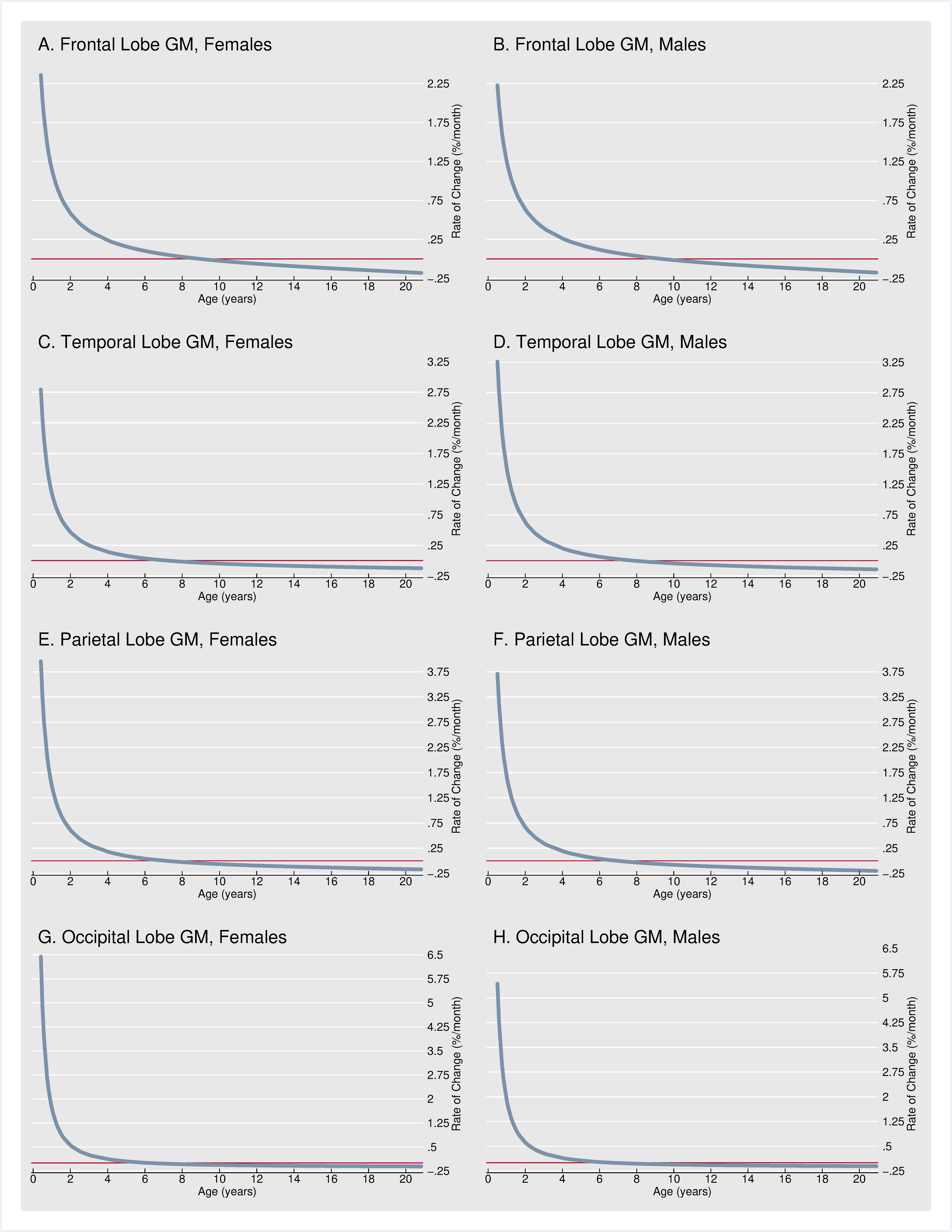

Supplement: S6 Fig — In S6 Fig, we plot the estimated relative growth rate (%/month) for GM in the frontal, temporal, parietal, and occipital lobes. Relative growth rates are expressed as a percentage of the predicted lobe size at the same age. (TIF) [file pone.0262607.s006.tif]

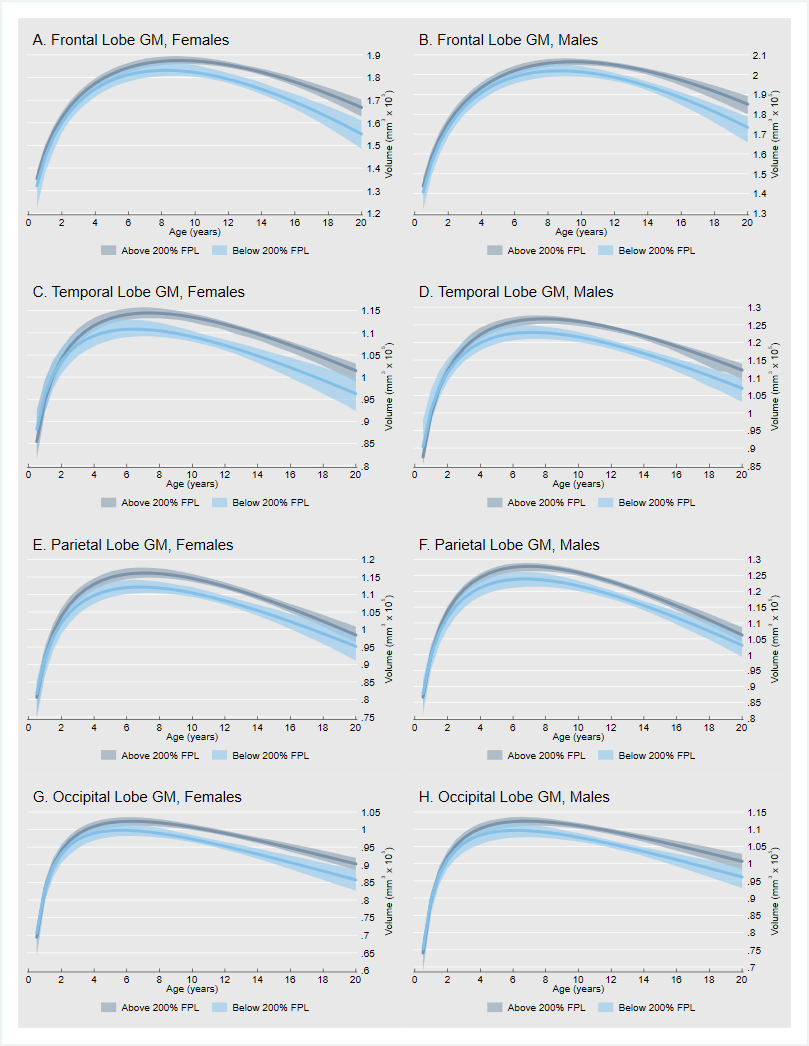

Supplement: S7 Fig — shows modeled GM volume trajectories for the frontal, temporal, parietal, and occipital lobes by household income. Gray lines outline typical GM development in children from nonpoor families with household income above 200% of the federal poverty level (FPL). Blue lines outline GM development in children from poor and near poor families with household income below 200% of the FPL. Models include birth weight, an indicator of both early health status and initial head size, as a covariate. (TIF) [file pone.0262607.s007.tif]

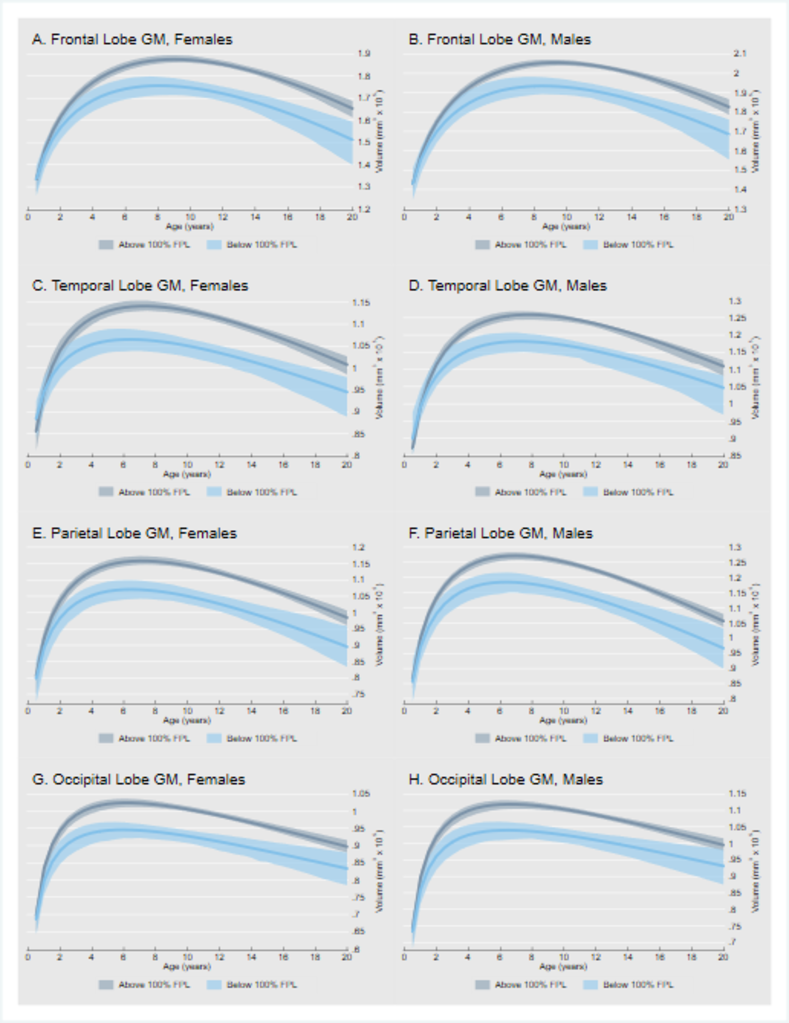

Supplement: S8 Fig — shows modeled GM volume trajectories for the frontal, temporal, parietal, and occipital lobes by household income. Gray lines outline typical GM development in children from nonpoor families with household income above 100% of the federal poverty level (FPL). Blue lines outline GM development in children from poor families with household income below 100% of the FPL. (TIF) [file pone.0262607.s008.tif]
