# Supplementary material for: Low household income and neurodevelopment from infancy through adolescence
Source: PLoS One. 2022 Jan 26;17(1):e0262607. doi: 10.1371/journal.pone.0262607 (PMC8791534; doi:10.1371/journal.pone.0262607)
Supplement: S1 File — (DOCX) [file pone.0262607.s009.docx]

**Statistical appendix**

**Modelling normal brain development**

Consistent with prior longitudinal work, mixed effect linear models (LMM) were used to assess GM volume growth trajectories in each of the brain’s four lobes.

$${volume}_{iSt}=f\left( {sex}_{i},{age}_{it} \right)+u_{S}+v_{i}+\in_{it}$$

Estimated trajectories allow for sex differences in both level and growth. To account for potential variation across study centers we fit a three-level model with a random intercept at both the study center ($u_{S}$) and individual ($v_{i}$) levels. Robust standard errors allow for heteroskedasticity with respect study center.

Lobular GM volumes follow an `inverted-U’ shaped trajectory. An initial period of growth is followed by a period of pruning as the brain cuts off unused pathways. The pace of growth is particularly rapid in infancy, with the brain nearly doubling in size within the first year of life. In work with children and adolescents the fixed component of LMM is most commonly modelled using a quadratic function of age. In order to capture the shape and pace of development across a broad age span (age 5 mo. to 21 y.) the fixed component, a function of sex and age (mo.), was modelled using fractional polynomials (FP).

Model selection in the context of LMM is complicated by the fact that a candidate FP transformation $f\left( {sex}_{i},{age}_{it} \right)$ may influence both the fixed effects and random effects structures. We pursued an established approach for utilizing fractional polynomials to model non-linear trends in longitudinal data: a small number of random effects ($u_{S}+v_{i}$) were held constant while assessing the influence of various fractional polynomial transformations on measures of global fit.

**Step-up selection process**

1. Select best fitting FP (second-order FP)
2. Compare to conventional polynomial (CP) of same order (quadratic)
3. If FP has superior fit, compare to CP of next highest order (cubic)
4. etc.

Best-fit trajectories, i.e., FP transformations for the fixed component $f\left( {sex}_{i},{age}_{it} \right)$, were determined for each region of interest based on measures of global fit (-2LL, AIC, BIC). The selected FP transformations are outlined below.

**Frontal lobe: FP(0.5, 1)**

$b_{0}+b_{1}\left( {age}_{it} \right)^{0.5}+b_{2}{age}_{it}+{male}_{i} \left[ b_{3}+b_{4}\left( {age}_{it} \right)^{0.5}+b_{5}{age}_{it} \right]$

**Temporal and parietal lobes: FP(0.5, 0.5)**

$$b_{0}+b_{1}\left( {age}_{it} \right)^{0.5}+b_{2}\left( {age}_{it} \right)^{0.5}ln\left( {age}_{it} \right)+{male}_{i} \left[ b_{3}+b_{4}\left( {age}_{it} \right)^{0.5}+b_{5}\left( {age}_{it} \right)^{0.5}ln\left( {age}_{it} \right) \right]$$

**Occipital lobe: FP(0, 0.5)**

$$b_{0}+b_{1}ln\left( {age}_{it} \right)+b_{2}\left( {age}_{it} \right)^{0.5}+{male}_{i} \left[ b_{3}+b_{4}ln\left( {age}_{it} \right)+b_{5}\left( {age}_{it} \right)^{0.5} \right]$$

Finally, for each region of interest, we tested (and rejected) additional random effects.

**Rate of growth**

Rates of lobular GM growth were estimated by taking the first derivative (with respect to age) of the corresponding GM volume trajectory. The resulting equations for growth are outlined below.

**Frontal lobe**

$$0.5 b_{1}\left( {age}_{it} \right)^{-0.5}+b_{2}+{male}_{i} \left[ 0.5 b_{4}\left( {age}_{it} \right)^{-0.5}+b_{5} \right]$$

**Temporal and parietal lobes**

$[0.5 b_{1}{+b_{2} \left( 1+0.5 ln\left( {age}_{it} \right) \right)] \left( {age}_{it} \right)}^{-0.5}+ {male}_{i} [0.5 b_{4}{+b_{5} \left( 1+0.5 ln\left( {age}_{it} \right) \right)] \left( {age}_{it} \right)}^{-0.5}$

**Occipital lobe**

$$b_{1}\left( {age}_{it} \right)^{-1}+{0.5 b}_{2}\left( {age}_{it} \right)^{-0.5}+{male}_{i} \left[ b_{4}\left( {age}_{it} \right)^{-1}+{0.5 b}_{5}\left( {age}_{it} \right)^{-0.5} \right]$$

**Volume-for-Age Percentiles**

For each of the brain’s lobes, we calculated individual residuals for each data point using the estimated GM volume trajectories.

$$x_{i}-\bar{x}={vol}_{iSt}- E\left[ {vol}_{iSt}| {sex}_{i},{age}_{it} \right]= {vol}_{iSt}- \hat{f\left( {sex}_{i},{age}_{it} \right)}- u_{s}$$

Squared residuals $\left( x_{i}-\bar{x} \right)^{2}$ are modelled as a function of age. Based on measures of global fit, we utilized first-order fractional polynomial models (though the squared residuals are nearly constant with age). The selected FP transformations are outlined below.

**Frontal lobe**

$$\left( x_{i}-\bar{x} \right)^{2}= b_{0}+b_{1}\ln\left( {age}_{it} \right)+ {male}_{i}\left[ b_{2}+b_{3}\ln\left( {age}_{it} \right) \right]$$

**Temporal and parietal lobes**

$$\left( x_{i}-\bar{x} \right)^{2}= b_{0}+b_{1}{{age}_{it}}^{-0.5}+ {male}_{i}\left[ b_{2}+b_{3}{{age}_{it}}^{-0.5} \right]$$

**Occipital lobe**

$$\left( x_{i}-\bar{x} \right)^{2}= b_{0}+b_{1}{{age}_{it}}^{-2}+ {male}_{i}\left[ b_{2}+b_{3}{{age}_{it}}^{-2} \right]$$

These best-fit models provide estimates of the population standard deviation (a function of sex and age) in lobular GM volume that are ultimately used to calculate sex-specific volume-for-age curves.

$$SD= \sqrt{\frac{\sum\left( x_{i}-\bar{x} \right)^{2}}{n}} \approx\sqrt{E\left[ \left( x_{i}-\bar{x} \right)^{2} \right]}$$

$$P_{A}=\bar{x}+z_{\left( \frac{100-A}{100} \right)}SD$$
